# Salvianolic acid B alleviates diabetic endothelial and mitochondrial dysfunction by down-regulating apoptosis and mitophagy of endothelial cells

**DOI:** 10.1080/21655979.2022.2026552

**Published:** 2022-01-22

**Authors:** Jie Xiang, Chunling Zhang, Tietao Di, Lu Chen, Wei Zhao, Lianggang Wei, Shiyong Zhou, Xueli Wu, Gengxin Wang, Yun Zhang

**Affiliations:** a Monitoring Department, Guizhou Center for Disease Control and Prevention, Institute of Chronic Disease Prevention and Treatment, Guiyang, Guizhou, China; b Department of Nutrition, The Second Affiliated Hospital of Guizhou University of Traditional Chinese Medicine, Guiyang, Guizhou, China; c Department of Trauma Surgery, The Second Affiliated Hospital of Guizhou University of Traditional Chinese Medicine, Guiyang, Guizhou, China; d Department of Endocrinology, The Second Affiliated Hospital of Guizhou University of Traditional Chinese Medicine, Guiyang, Guizhou, China; e Department of Rheumatology, Chongqing Traditional Chinese Medicine Hospital, Chongqing, China; f Department of General Surgery, The Second Affiliated Hospital of Guizhou University of Traditional Chinese Medicine, Guiyang, Guizhou, China; g Central Laboratory, The Second Affiliated Hospital of Guizhou University of Traditional Chinese Medicine, Guiyang, Guizhou, China; h Graduate School, Guizhou University of Traditional Chinese Medicine, Guiyang, Guizhou, China

**Keywords:** Salvianolic acid B, endothelial dysfunction, diabetes, mitophagy, apoptosis

## Abstract

Endothelial dysfunction is a critical mediator in the pathogenesis of vascular complications of diabetes. Herein, this study was conducted to investigate the therapeutic effects of Salvianolic acid B (Sal B) on diabetes-induced endothelial dysfunction and the underlying mechanisms. Diabetic models were established both in db/db mice and high glucose (HG)-induced human umbilical vein endothelial cells (HUVECs). Moreover, HUVECs were exposed to carbonyl cyanide m-chlorophenyl hydrazone (CCCP) to induce endothelial cell damage. Following Sal B treatment, pathological changes of thoracic aorta were investigated by hematoxylin and eosin, alcian blue (AB), elastic fiber, Masson, and reticular fiber staining. BCL2-associated X (BAX), B-cell lymphoma-2 (Bcl-2), Beclin1, Parkin and PTEN Induced Kinase 1 (Pink1) expression was detected by Western blot, immunohistochemistry, and immunofluorescence in thoracic aorta, HG- and CCCP-induced HUVECs. Cell scratch test, MitoTracker Red CMXRos staining and Flou-4 AM staining were separately presented to detect migration, mitochondrial activity and intracellular Ca^2+^ in HUVECs. Our results showed that Sal B significantly ameliorated hyperlipidemia, hyperglycemia, hyperinsulinemia, and insulin resistance in db/db mice. Furthermore, it significantly alleviated diabetes-induced vascular endothelial dysfunction according to histopathology analysis. In diabetic thoracic aorta, HG- and CCCP-induced HUVECs, Sal B distinctly increased Bcl-2 expression and reduced BAX, Beclin1, Parkin and Pink1 expression, thereby protecting endothelial cells from apoptosis and mitophagy. Moreover, Sal B markedly enhanced migration, mitochondrial activity and intracellular Ca^2+^ levels both in HG- and CCCP-induced HUVECs. Collectively, Sal B exhibited a potential to improve diabetes-induced endothelial and mitochondrial dysfunction through down-regulating apoptosis and mitophagy of endothelial cells.

**Abbreviations:** DM: diabetes mellitus; T2DM: type 2 diabetes mellitus; Sal B: Salvianolic acid B; HG: high glucose; FBG: fasting blood glucose; TC: total cholesterol; TG: triglycerides; LDL-C: low-density lipoprotein cholesterol; HDL-C: high-density lipoprotein cholesterol; FINS: fasting insulin; HOMA-IR: homeostasis model assessment insulin resistance; QUICKI: quantitative insulin-sensitivity check index; H&E: hematoxylin and eosin; HUVECs: human umbilical vein endothelial cells; IHC: immunohistochemistry; CCCP: carbonyl cyanide m-chlorophenyl hydrazone; FCM: flow cytometry; CCK-8: cell counting kit-8

## Introduction

Diabetes mellitus (DM) is gradually becoming a global public health concern [1]. Type 2 diabetes mellitus (T2DM) is the most prevalent type of diabetes, accounting for about 90–95% of all diabetes cases globally [2]. T2DM is characterized by progressive insulin deficits and β-cell dysfunction and insulin resistance [3]. Cardiovascular complications contribute to deaths in 68% of patients with type 2 diabetes [4]. Endothelial cells line up in the internal blood vessels, which regulate blood vessel tension and maintain blood vessel homeostasis [5]. Endothelial dysfunction in both macro- and micro-vascular complications is a key event in the onset and progression of T2DM-induced vascular complications [6]. Hence, it is of importance to clarify the molecular mechanisms underlying diabetes-induced endothelial dysfunction and to exploit more effective drugs.

In diabetic patients, endothelial cells exhibit mitochondrial damage, mitochondrial dysfunction, and reduced oxidative ability [7]. Mitochondria, double-membrane-bound organelles, play a key role in energy production as well as cell homeostasis [8]. Mitochondrial dysfunction has an association with mitochondrial calcium overload that induces decreased mitochondrial membrane potential, damaged mitochondrial respiration as well as excessive production of mitochondrial reactive oxygen species, thereby contributing to endothelial injury in diabetic conditions [9–11]. Mitophagy describes the signaling cascade mitochondria are degraded through autophagy and impaired mitophagy induces the progression of diabetic endothelial dysfunction [12]. Thus, targeting mitochondrial dysfunction represents an attractive strategy to alleviate diabetic vascular diseases.

Salvianolic acid B (Sal B) is a natural bioactive antioxidant derived from Radix Salvia miltiorrhiza [13]. It has been popularly applied for the treatment of cardiovascular and cerebrovascular diseases [14]. Especially, Sal B has several therapeutic effects on insulin resistance, diabetes, and obesity [15–17]. Furthermore, limited experimental evidence has suggested that Sal B can ameliorate endothelial dysfunction. For instance, Sal B alleviates oxidized low-density lipoprotein- and high glucose (HG)-induced endothelial dysfunction through weakening ROCK1-mediated mitophagy and apoptosis [18]. Sal B alleviates vascular endothelial dysfunction for diabetic rats through suppressing endothelial cell apoptosis [15]. Nevertheless, the molecular mechanisms of Sal B on diabetes-induced endothelial dysfunction remain indistinct. Herein, this study aimed to investigate the therapeutic effects of Sal B on diabetes-induced endothelial dysfunction and the underlying mechanisms. We hypothesized that Sal B alleviated diabetes-induced endothelial dysfunction through down-regulating apoptosis and mitophagy of endothelial cells. Our findings indicated that Sal B possessed the potential as a therapeutic agent against diabetic endothelial and mitochondrial dysfunction. More studies will be conducted to further confirm the therapeutic effects of Sal B on diabetes-induced endothelial dysfunction in our future studies.

## Materials and methods

### Animals and experimental design

7-week-old male diabetic C57BL/KsJ db/db mice (35–40 g) and age-matched non-diabetic C57BL/KsJ db/m mice with 7-week-old and 16–18 g were purchased from Changzhou cavens experimental animal Co., Ltd (http://www.cavens.com.cn/). These animals were raised at 22 ± 2°C in an atmosphere of 60 ± 5% relative humidity and a 12 h light/dark cycle. The animal experimental procedures strictly followed the Guide for the Care and Use of Laboratory Animals of the National Institutes of Health. This project gained the approval of the Animal Care and Use Committee of the Second Affiliated Hospital of Guizhou University of Traditional Chinese Medicine (GK2019010). Diabetes was confirmed if fasting blood glucose level >16.7 mM. The mice were randomly separately into three groups (15 mice/group): control group (db/m mice); model group (db/db mice) and Sal B group (db/db mice). All mice were fed a commercial diet and allowed free access to water and food. Sal B was purchased from Med Chem Express Company (HY-N1362; www.medchemexpress.cn), with a purity 99.93% by high performance liquid chromatography (HPLC). For the Sal B group, db/db mice were orally treated with 50 mg/kg Sal B each day for 6 weeks. The dose of Sal B was determined according to a published study [19]. Mice in the control and model groups were orally administered with the equal amount of saline. All mice were euthanized by 5% isoflurane. Thoracic aorta was dissected from each mouse, instantly frozen in liquid nitrogen, and stored at −80°C.

### Detection of serum biochemical indicators

After 6 weeks of treatment, serum biochemical indicators were measured. After 24 h fasting, 1 ml blood was collected from the tail vein of each mouse. Fasting blood glucose (FBG) was tested through Glucose Assay Kit (F006-1-1; Nanjing Jiancheng Bioengineering Institute, China). Then, whole blood was harvested and serum was isolated for biochemical measurement. Through automatic biochemical analyzer, total cholesterol (TC), triglycerides (TG), low-density lipoprotein cholesterol (LDL-C) and high-density lipoprotein cholesterol (HDL-C) were detected in serum specimens. Fasting insulin (FINS) was examined Insulin Assay Kit (H203-1-1; Nanjing Jiancheng Bioengineering Institute, China). Homeostasis model assessment insulin resistance (HOMA-IR) was calculated following the formula: HOMA-IR = FBG (mmol/L) × FINS (mIU/L)/22.5 [20]. Furthermore, insulin resistance was also evaluated with quantitative insulin-sensitivity check index (QUICKI) according to the formula: QUICKI = 1/[log(FINS) + log(FBG)] [21].

### Histopathology analysis

Thoracic aorta tissues were fixed in 4% paraformaldehyde, embedded in paraffin and sectioned at 5 µm. The sections were deparaffinized to water, followed by being stained with hematoxylin and eosin (H&E; G1120; Solarbio, Beijing, China), alcian blue (AB; BA4121; BASO, Wuhan, China), elastic fiber (BA4083A; BASO, Wuhan, China), Masson (BA4079A; BASO, Wuhan, China) as well as reticular fiber (BA4165; BASO, Wuhan, China). For H&E staining, the sections were stained by hematoxylin lasting 5 min and differentiated by hydrochloric acid ethanol at room temperature for 3 s. After returning to blue by rinsing with tap water for 30 min, the sections were counter-stained with eosin staining solution for 2 min. For AB staining, the slices were stained by alcian blue lasting 20 min. Afterward, the sections were oxidized with 0.5% periodic acid aqueous solution for 10 min, and stained with Schiff reagent for 15 min. For elastic fiber staining, the sections were incubated with Lugol iodine solution lasting 5 min and treated by sodium thiosulfate lasting 5 min. Subsequently, the sections were stained with aldehyde fuchsin staining solution for 10 min. For Masson staining, the sections were stained with Weigert’s iron hematoxylin for 5 min. After a few seconds of differentiation with 1% hydrochloric acid ethanol, the sections were washed with tap water for 30 min and turned blue. The slices were stained by ponceau acid fuchsin solution lasting 10 min. Following being treated by phosphomolybdic acid aqueous solution lasting 5 min, the slices were counterstained by aniline blue solution lasting 5 min as well as treated by 1% glacial acetic acid for 1 min. For reticular fiber staining, the sections were oxidized with 1% potassium permanganate lasting 5 min as well as treated by 2% oxalic acid lasting 2 min and 1% uranium nitrate for 10 sec, followed by being immersed in ammonia silver solution for 1 min. After washing with 95% alcohol, the sections were developed in the developer for 1 min. Subsequently, the sections were tinted with 0.2% gold chloride for 1 min and treated with 5% sodium sulfite for 1 min. After dehydration with gradient ethanol, the sections were mounted with transparent xylene, followed by neutral gum. Finally, the sections were investigated under a light microscope (Nikon, Japan).

### HUVEC culture and treatment

Human umbilical vein endothelial cells (HUVECs; ATCC, USA) were grown in Dulbecco’s modified Eagle’s medium (Gibco, USA) plus 10% fetal bovine serum (FBS), 100 U/ml penicillin and 100 U/ml streptomycin. HUVECs were grown in an environment of 5% CO_2_ at 37°C. Diabetes cell model was constructed by 30 mM high-glucose (HG)-induced HUVECs. 0.1% DMSO was utilized for improving the solubility and bioavailability of Sal B. HUVECs were treated with 0, 5, 10, 30, 50, 80 and 100 μM Sal B for 48 h in HG-pre-treated HUVECs. Furthermore, HUVECs were treated with 50 nM oxidative phosphorylation uncoupler carbonyl cyanide m-chlorophenyl hydrazone (CCCP; HY-100941; Med Chem Express, USA) that was dissolved by 0.1% DMSO.

### Western blot

Thoracic aorta tissues or HUVECs were lysed through lysis buffer (20 mM Tris-hydrochloric acid (pH = 7.5), 137 mM sodium chloride, 0.5% ethyl phenyl polyethylene glycol, 0.5 mM dithiothreitol, complete protease inhibitor cocktail, and phosphatase inhibitor). Bradford protein concentration determination kits were applied for determining the concentration of total protein. The protein was separated by 10% SDS-PAGE and transferred to PVDF membrane (Bio-Rad, USA). The membrane was incubated with primary antibody (anti-BCL2-associated X (anti-BAX; 1/1000; ab53154; Abcam, USA), anti-B-cell lymphoma-2 (anti-Bcl-2; 1/1000; ab194583; Abcam, USA), anti-Beclin1 (1/1000; 11306-1-AP; Proteintech, China), anti-Parkin (1/1000; 14060-1-AP; Proteintech, China), anti-PTEN Induced Kinase 1 (anti-Pink1 (1/1000; 23274-1-AP; Proteintech, China) and anti-β-actin (1/1000; 66009-1-Ig; Proteintech, China)) overnight at 4°C. Afterward, the membrane was incubated by horseradish peroxidase-conjugated secondary antibody (1/2000; ab7090; Abcam, USA) lasting 1 h at room temperature. The protein band was developed with enhanced chemiluminescence kits (Thermo Fisher Scientific, USA), followed by quantification through Image-Pro Plus 6.0 software (National Institutes of Health).

### Immunohistochemistry (IHC) and immunofluorescence

HUVECs were planted onto a 6-well plate (1 × 10^5^ cells/well), followed by glass coverslips for 24 h. Thoracic aorta tissues or HUVECs were fixed with 4% paraformaldehyde for 40 min on the ice. Afterward, thoracic aorta tissues or HUVECs were permeabilized by 0.1% Triton X-100 for 20 min and blocked by 5% BSA for 30 min. For immunohistochemistry, the sections were incubated with primary antibody lasting 90 min at 37°C. Primary antibodies included anti-BAX (1/100; ab53154; Abcam, USA), anti-Bcl-2 (1/100; ab194583; Abcam, USA), anti-Beclin1 (1/100; ab62557; Abcam, USA), anti-Parkin (1/100; 14060-1-AP; Proteintech, China) and anti-Pink1 (1/100; 23274-1-AP; Proteintech, China). Then, the sections were incubated by secondary antibodies (1:200; ab7090; Abcam, USA) for 30 min. The sections were stained by diaminobenzidine solution in the dark for 15 min, followed by being counterstained by hematoxylin for 10 min. For immunofluorescence, the sections were incubated by primary antibody (anti-BAX (1/100; ab53154; Abcam, USA), anti-Bcl-2 (1/100; ab194583; Abcam, USA), anti-Beclin1 (1/100; 11306-1-AP; Proteintech, China), anti-Parkin (1/100; 14060-1-AP; Proteintech, China) and anti-Pink1 (1/100; 23274-1-AP; Proteintech, China)) overnight at 4°C. Then, the sections were incubated by Alexa Fluor® 488 Conjugate (1:100; ZF-0512, ZSGB-BIO, China) or Alexa Fluor® 594 Conjugate (1:100; ZF-0513, ZSGB-BIO, China) secondary antibodies for 1 h at 37°C, followed by being stained with DAPI (Sigma, USA). Images were acquired utilizing a light microscope (Nikon, Japan) or a fluorescence microscope (Carl Zeiss, Germany).

### Flow cytometry (FCM)

Apoptotic levels were examined through Annexin V-FITC/PI double staining apoptosis detection kits (G003-1-2; Nanjing Jiancheng Bioengineering Institute, China). HUVECs were digested by trypsin and centrifugated for 10 min at 4°C. In the dark at 4°C, the cell suspension was stained by 5 µl Annexin V-FITC lasting 15 min as well as 10 µl PI lasting 5 min. Afterward, HUVECs were instantly detected through flow cytometer (BD, USA).

### Cell counting kit-8 (CCK-8)

CCK-8 (CK04; Dojindo, Japan) was applied to detect proliferation of HUVECs. HUVECs were added to a 96-well plate (2 × 10^3^ cells/well). Then, 10 μl CCK-8 solution was added to each well. HUVECs continued to incubate lasting 4 h at 37°C. Each test was set to 3 duplicate holes and repeated 3 times. The absorbance values at 450 nm were determined with a microplate reader.

### Cell scratch test

HUVECs were seeded onto a 6-well plate (1 × 10^6^ cells/well). After 24 h, HUVECs were grown to complete confluence and scratched by 10 µl pipette tips. Afterward, HUVECs were cultured by serum-free medium for 24 h. Images were acquired under a light microscope at 0 and 24 h. Cell scratch was assessed through measurement of the wound distance utilizing Image-Pro Plus 6.0 software.

### Measurement of mitochondrial activity

Mitochondria of HUVECs were stained by 100 nM MitoTracker Red CMXRos staining solution in the dark for 15 min. Images were captured by a fluorescence microscope (Carl Zeiss, Germany). Mitochondrial fluorescence intensity was quantified utilizing Image-Pro Plus 6.0 software.

### Flou-4 AM staining

Fluo-4 AM fluorescent probe (F14201; Invitrogen, USA) was utilized to detect intracellular Ca^2+^ levels. HUVECs were seeded onto a 6-well plate. In the dark, HUVECs were incubated by 2 μmol/L Fluo-4 AM for 30 min. Being washed twice with PBS, results were acquired under a fluorescence microscope (Carl Zeiss, Germany). The fluorescence intensity was quantified with Image-Pro Plus 6.0 software.

### Statistical analysis

Data are displayed as the mean ± standard deviation of ≥3 independent assays. Statistical analysis was presented with GraphPad Prism software (version 8.0.1). One-way analysis of variance followed by Tukey's test was applied for multi-group comparison. P < 0.05 indicated a statistical significance.

## Results

This study aimed to investigate the therapeutic effects of Sal B on diabetes-induced vascular endothelial dysfunction both in db/db mice and HG- and CCCP-induced HUVECs, and explored the underlying molecular mechanisms.

### Sal B ameliorates lipid, glucose, and insulin metabolism disorders in db/db mice

Here, db/db mice were used for establishing T2DM model. Following treatment of 50 mg/kg Sal B for 6 weeks, we measured serum lipid profiles including TC, TG, LDL-C and HDL-C by biochemical analysis. Our results showed that db/db mice displayed significantly increased serum TC, TG and LDL-C levels as well as reduced serum HDL-C levels than control mice (Table 1). However, we observed that 50 mg/kg Sal B markedly decreased serum TC, TG and LDL-C levels and elevated serum HDL-C levels in db/db mice. This study further observed the effects of Sal B on hyperglycemia for each db/db mouse. Compared to controls, db/db mice presented prominently increased FBG levels (Table 1). FBG levels of db/db mice were significantly lowered by 50 mg/kg Sal B treatment. Serum FINS levels were also measured. In Table 1, db/db mice presented hyperinsulinemia. Sal B administration markedly decreased serum FINS levels of db/db mice. Insulin resistance was evaluated through HOMA-IR and QUICKI indicators. Increased HOMA-IR and QUICKI values were observed in db/db mice than controls (Table 1). Following administration of 50 mg/kg Sal B for 6 weeks, HOMA-IR and QUICKI values were notably reduced in db/db mice. Collectively, db/db mice displayed hyperlipidemia, hyperglycemia, hyperinsulinemia, and insulin resistance. Sal B treatment markedly ameliorated lipid, glucose, and insulin metabolism disorders in db/db mice.

**Table 1.** Sal B ameliorates hyperlipidemia, hyperglycemia, hyperinsulinemia, and insulin resistance in db/db mice

Indicators	Control (n = 15)	Model (n = 15)	Sal B (n = 15)
TC	13.070480 ± 1.077478	18.030100 ± 1.467929****	15.299620 ± 1.066424^####^
TG	1.509515 ± 0.110713	2.578789 ± 0.485062****	1.800650 ± 0.233088^####^
LDL-C	1.245364 ± 0.390012	2.202455 ± 0.273423****	1.414636 ± 0.379273^####^
HDL-C	2.993992 ± 0.363342	1.385185 ± 0.259501****	2.217202 ± 0.239020^####^
FBG	5.340000 ± 0.605687	31.006670 ± 2.236536****	17.440000 ± 2.670687^####^
FINS	5.294200 ± 0.621903	15.364400 ± 1.716380****	10.529000 ± 1.624415^####^
HOMA-IR	1.253091 ± 0.180624	21.125300 ± 2.334503****	8.196887 ± 1.941515^####^
QUICKI	10.634200 ± 0.767940	46.371070 ± 2.373769****	27.969000 ± 3.390362^####^

Compared with control group, ****p < 0.0001; compared with model group, ^####^*p* < 0.0001.

### Sal B ameliorates diabetes-induced vascular endothelial dysfunction in db/db mice

**Figure 1.**
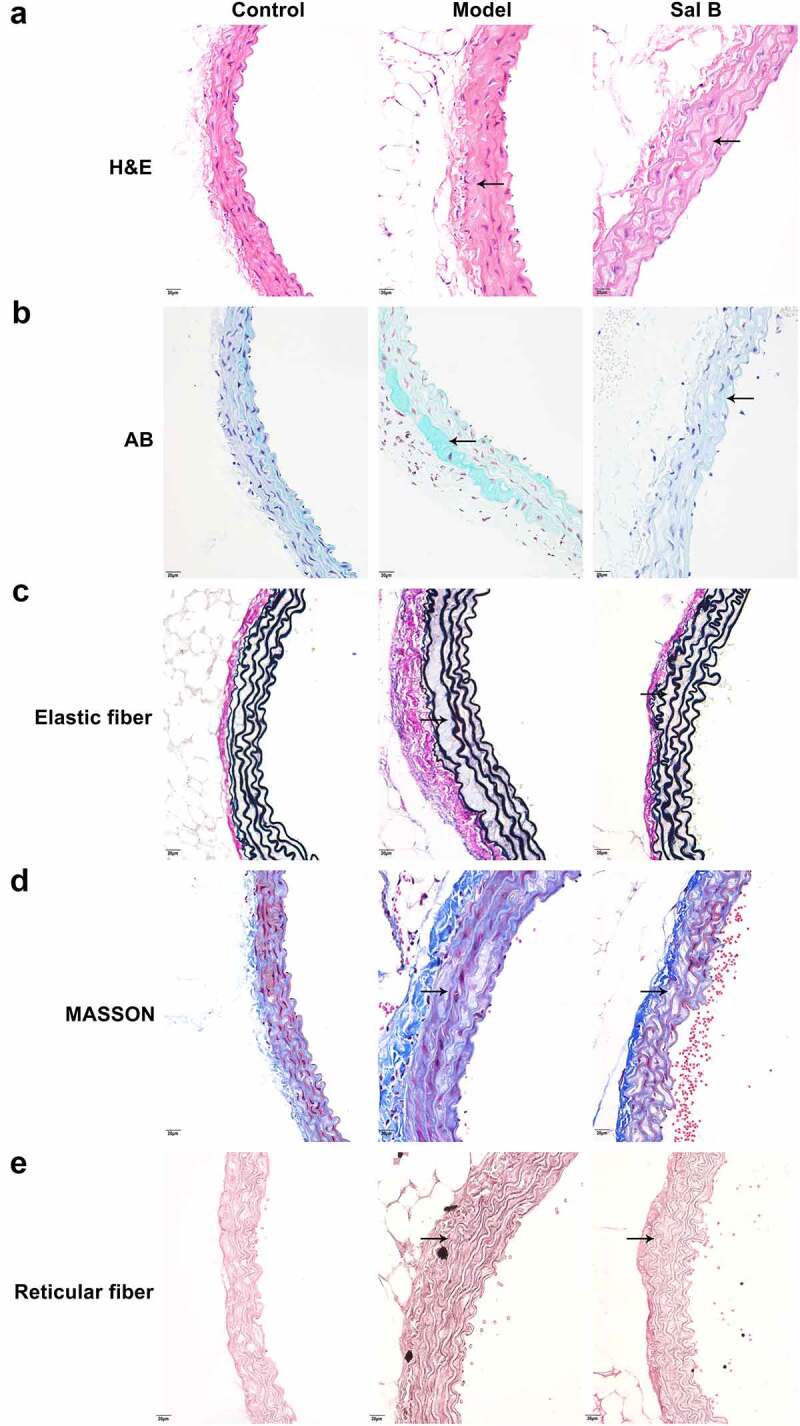
Sal B ameliorates diabetes-induced vascular pathology in db/db mice. (a–e) Representative images of (a) H&E staining, (b) AB staining, (c) elastic fiber staining (d) Masson staining, and (e) reticular fiber staining of thoracic aortas of db/m mice, db/db mice as well as db/db mice that were orally administrated 50 mg/kg Sal B for six weeks. Scale bar, 20 μm. Arrows indicate the abnormalities as claimed.

H&E staining showed that compared with the control group, the arterial wall was significantly thickened, the arterial media and adventitia space was widened, and infiltrating cells displayed significant increase in the model group (Figure 1(a)). As shown by AB staining, compared to the control mice, blue acidic mucus was seen in the arterial media and adventitia space of the db/db model mice (Figure 1(b)). Masson staining showed that the collagen fibers of the model group were obviously thicker, and the cell arrangement was chaotic and unevenly distributed in comparison to the control group (Figure 1(c)). According to elastic fiber staining results, compared to controls, the arterial media as well as adventitia gaps were widened and lost the original uneven shape in the model group (Figure 1(d)). Reticular fiber staining showed that, in comparison to the control mice, the reticular fiber staining of the db/db mice was enhanced and disordered (Figure 1(e)). However, Sal B administration markedly improved the pathological changes of the thoracic aorta in spontaneous type 2 diabetic db/db mice by relieving the thickening of the vascular wall and reducing the infiltrating cells of the arterial media and adventitia (Figure 1(a)); lowering the exudation of acidic fluid in the vascular tissue (Figure 1(b)); reducing collagen fibers (Figure 1(c)); increasing the stretchability of vascular elastic fibers (Figure 1(d)); decreasing the density of reticular fibers (Figure 1(e)).

### Sal B protects against apoptosis and mitophagy in thoracic aorta of db/db mice

**Figure 2.**
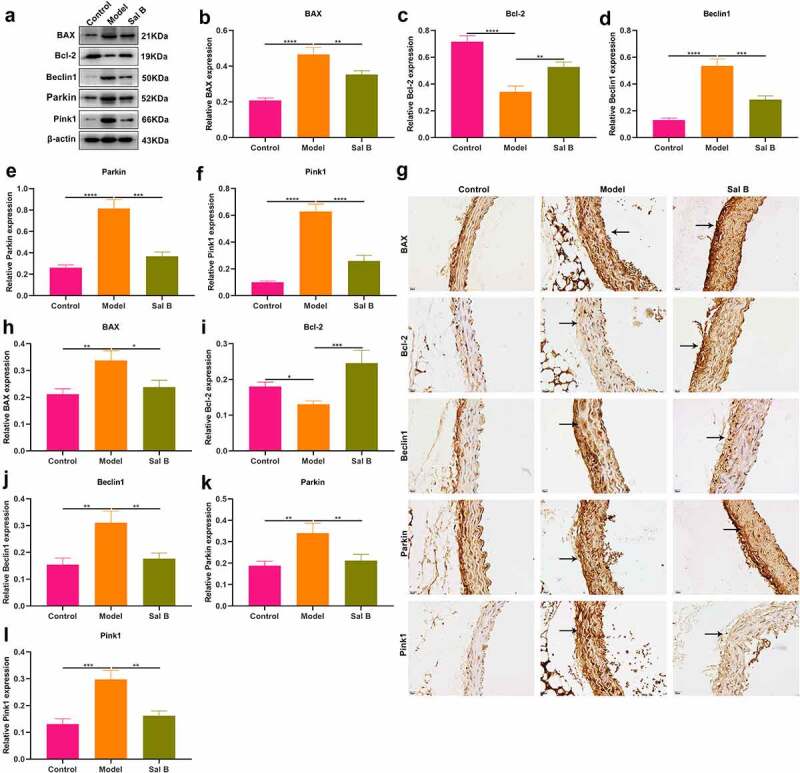
Sal B protects against apoptosis and mitophagy in thoracic aortas of db/db mice. (a–f) Western blot detecting BAX, Bcl-2, Beclin1, Parkin, and Pink1 expression in thoracic aortas of db/m mice, db/db mice as well as db/db mice that were orally administrated 50 mg/kg Sal B for six weeks. (g–l) IHC staining of BAX, Bcl-2, Beclin1, Parkin, and Pink1 expression in thoracic aortas of above mice. Scale bar, 20 μm. *P < 0.05; **p < 0.01; ***p < 0.001; ****p < 0.0001. Arrows indicate the abnormalities as claimed.

**Figure 3.**
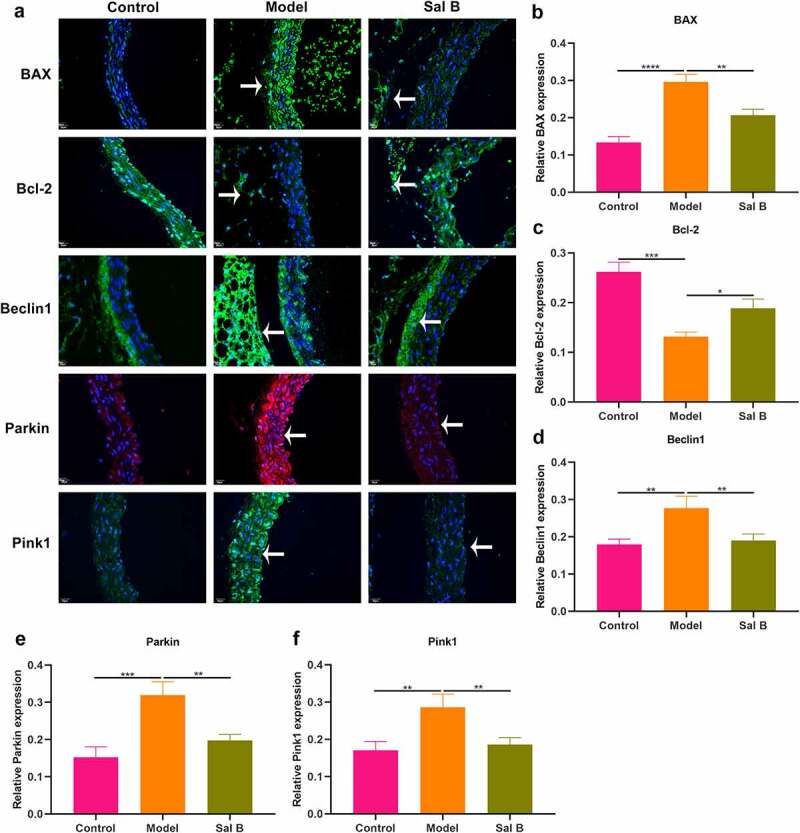
Effects of Sal B on apoptosis and mitophagy in thoracic aortas of db/db mice. (a) Representative images of immunofluorescence of BAX, Bcl-2, Beclin1, Parkin, and Pink1 in thoracic aortas from db/m mice, db/db mice as well as db/db mice that were orally administrated 50 mg/kg Sal B for 6 weeks. Scale bar, 20 μm. Arrows indicate the abnormalities as claimed. (b–f) Quantification of the expression of BAX, Bcl-2, Beclin1, Parkin, and Pink1 in thoracic aorta of above mice. *P < 0.05; **p < 0.01; ***p < 0.001; ****p < 0.0001.

The effects of Sal B on apoptosis were observed in thoracic aorta of db/db mice through detection of apoptosis markers BAX and Bcl-2. Western blot showed that db/db mice presented significantly elevated expression of BAX as well as significantly reduced expression of Bcl-2 in thoracic aorta (Figure 2(a–c)). However, Sal B administration markedly weakened BAX expression and elevated Bcl-2 expression in thoracic aorta of db/db mice. Mitochondrial injury and dysfunction induce vascular endothelial dysfunction. Beclin1 and Pink1/Parkin signaling may activate mitophagy. Here, we observed that Beclin1, Parkin and Pink1 displayed significantly increased expression in thoracic aorta of db/db mice, suggesting the activation of mitophagy (Figure 2(d–f)). Following Sal B administration for 6 weeks, Beclin1, Parkin and Pink1 expression was all markedly reduced in thoracic aorta of db/db mice. IHC staining was also presented for detecting the expression of BAX, Bcl-2, Beclin1, Parkin as well as Pink1 (Figure 2(g)). Consistent with the results of Western blots, BAX expression was markedly elevated as well as Bcl-2 expression was prominently weakened in thoracic aorta of db/db mice than controls (Figure 2(h,i)). Sal B administration alleviated BAX expression and activated Bcl-2 expression in thoracic aorta of db/db mice. Activation of Beclin1, Parkin and Pink1 expression was found in diabetic thoracic aorta, which was alleviated by Sal B treatment (Figure 2(j–l)). To further verify the anti-apoptosis and anti-mitophagy effects of Sal B in db/db mice, we carried out immunofluorescence. Consistently, Sal B ameliorated diabetes-induced apoptosis in thoracic aorta by reducing BAX expression and elevating Bcl-2 expression (Figure 3(a–c)). Furthermore, Sal B alleviated diabetes-induced mitophagy in thoracic aorta by decreasing Beclin1, Parkin and Pink1 expression (Figure 3(d–f)).

### Sal B enhances migration and mitochondrial activity and alleviates apoptosis and mitophagy in HG-induced HUVECs

**Figure 4.**
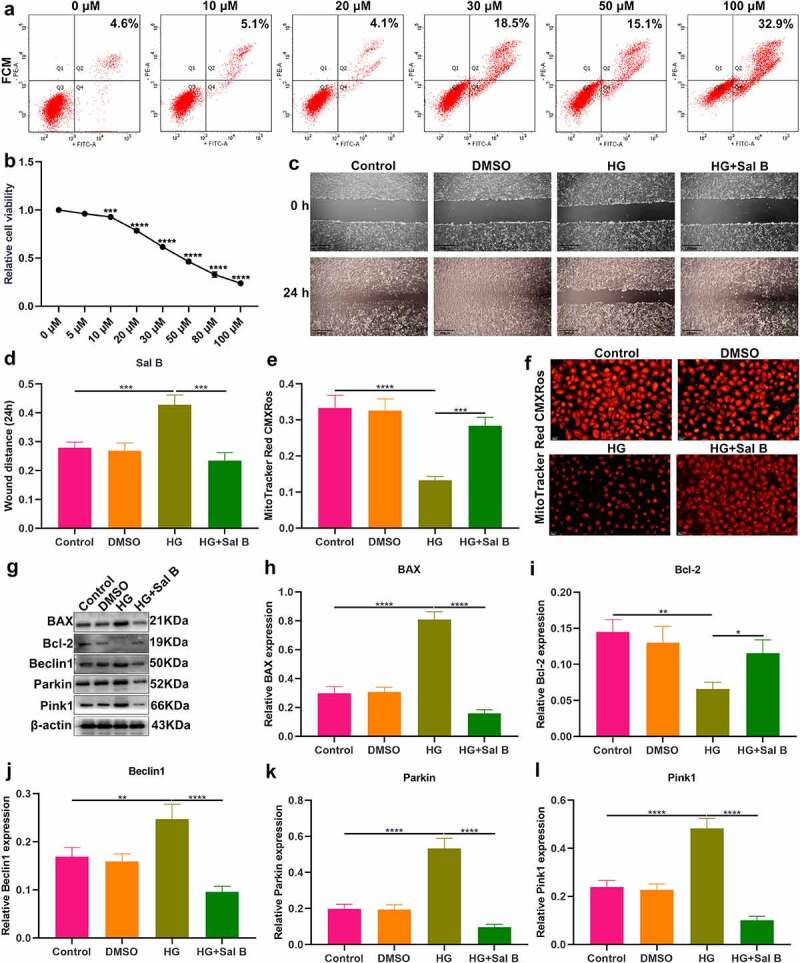
Sal B enhances migration and mitochondrial activity and alleviates apoptosis and mitophagy in HG-induced HUVECs. (a) FCM detecting the apoptosis of HUVECs treated by 0, 10, 20, 30, 50 and 100 μM Sal B. (b) CCK-8 examining the cell viability of HUVECs treated by 0, 5, 10, 20, 30, 50, 80 and 100 μM Sal B. (c, d) Cell scratch test detecting the wound distance of HUVECs in four groups: control, DMSO, HG and HG + Sal B. Scale bar, 200 μm. (e, f) Detection of mitochondrial activity of HUVECs in above groups through MitoTracker Red CMXRos staining. Scale bar, 20 μm. (g–l) Western blot detecting the expression of BAX, Bcl-2, Beclin1, Parkin and Pink1 in HUVECs of above groups. *P < 0.05; **p < 0.01; ***p < 0.001; ****p < 0.0001.

**Figure 5.**
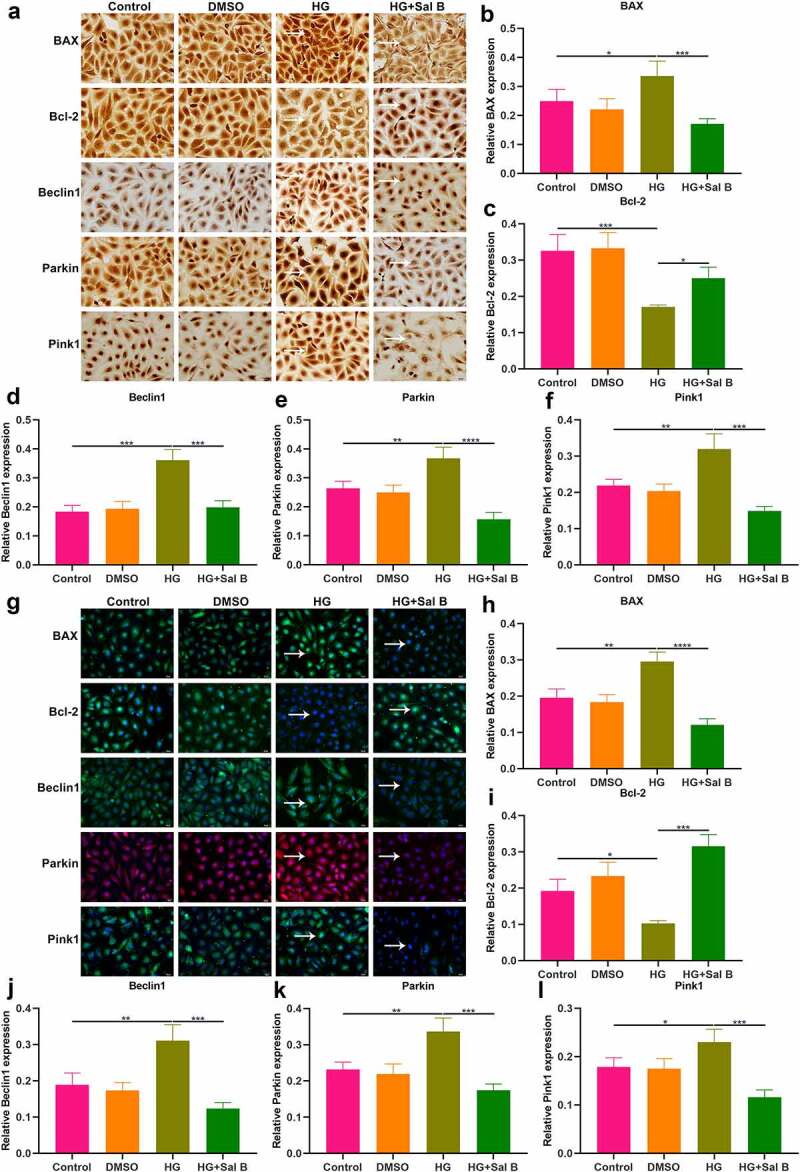
Sal B alleviates apoptosis and mitophagy in HG-induced HUVECs. (a–f) IHC detecting the expression of BAX, Bcl-2, Beclin1, Parkin and Pink1 in HUVECs of four groups: control, DMSO, HG and HG + Sal B. Scale bar, 20 μm. (g–l) Immunofluorescence examining the expression of BAX, Bcl-2, Beclin1, Parkin and Pink1 in HUVECs of above groups. Scale bar, 20 μm. *P < 0.05; **p < 0.01; ***p < 0.001; ****p < 0.0001. Arrows indicate the abnormalities as claimed.

We further observed the effects of Sal B on HG-induced HUVECs. By FCM and CCK-8, 20 μM was identified as the optimal dose of Sal B in HUVECs (Figure 4(a,b)). We observed that HG-induced HUVECs presented prominently weakened migration ability than controls (Figure 4(c,d)). Following Sal B treatment, migration ability of HG-induced HUVECs was markedly ameliorated. To observe the effect of Sal B on mitochondrial activity of HUVECs, MitoTracker Red CMXRos staining was presented. We observed that fluorescence intensity of mitochondria was markedly reduced in HG-induced HUVECs than controls (Figure 4(e,f)). However, Sal B treatment significantly elevated fluorescence intensity of mitochondria in HG-induced HUVECs. Apoptosis markers BAX and Bcl-2 were examined to evaluate the apoptotic levels of HUVECs. We found that BAX expression displayed significant increase and Bcl-2 expression exhibited distinct decrease in HG-induced HUVECs, suggesting that HG significantly enhanced apoptosis of HUVECs (Figure 4(g–i)). As expected, Sal B treatment substantially lowered BAX expression as well as elevated Bcl-2 expression in HG-induced HUVECs. Through Western blot, mitophagy was evaluated by measuring the expression of Beclin1, Parkin and Pink1. We observed that HG treatment markedly enhanced the expression of Beclin1, Parkin and Pink1 in HUVECs, suggesting the activation of mitophagy (Figure 4(j–l)). Their expression was all distinctly reduced by Sal B treatment in HG-induced HUVECs. IHC staining was utilized for measurement of the expression of BAX and Bcl-2. Consistent with the results of Western blot, HG-induced HUVECs exhibited a significant increase in BAX expression as well as a distinct reduction in Bcl-2 expression than controls (Figure 5(a–c)). Sal B treatment markedly reduced BAX expression and elevated Bcl-2 expression in HG-induced HUVECs. Meanwhile, Beclin1, Parkin and Pink1 expression displayed the distinct increase in HG-induced HUVECs, which was significantly alleviated by Sal B (Figure 5(d–f)). Immunofluorescence also showed the apoptosis induced by HG in HUVECs according to highly expressed BAX and lowly expressed Bcl-2 (Figure 5(g–i)). Sal B treatment prominently suppressed apoptosis of HG-induced HUVECs. Moreover, mitophagy was distinctly enhanced in HUVECs induced by HG, which was alleviated by Sal B treatment (Figure 5(j–l)). Collectively, above findings suggested that Sal B enhanced migration and mitochondrial activity as well as alleviated apoptosis and mitophagy in HG-induced HUVECs.

### Sal B enhances migration and mitochondrial activity of CCCP-induced HUVECs and increases intracellular Ca^2+^ levels both in HG- and CCCP-induced HUVECs

**Figure 6.**
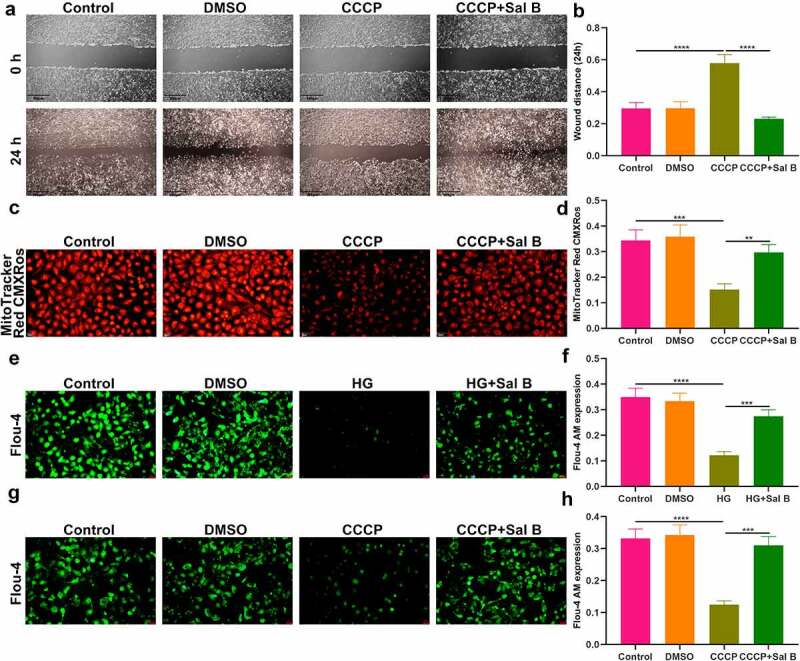
Sal B enhances migration and mitochondrial activity of CCCP-induced HUVECs and increases intracellular Ca^2+^ levels both in HG- and CCCP-induced HUVECs. (a, b) Cell scratch test detecting the wound distance of HUVECs in four groups: control, DMSO, CCCP and CCCP + Sal B. Scale bar, 200 μm. (c, d) Measurement of mitochondrial activity of HUVECs in above groups through MitoTracker Red CMXRos staining. Scale bar, 20 μm. (e, f) Detection of intracellular Ca^2+^ levels of HUVECs in control, DMSO, HG and HG + Sal B groups. Scale bar, 20 μm. (g, h) Detection of intracellular Ca^2+^ levels of HUVECs in control, DMSO, CCCP and CCCP + Sal B groups. Scale bar, 20 μm. **P < 0.01; ***p < 0.001; ****p < 0.0001.

It has been confirmed that CCCP can induce mitochondrial dysfunction and damage [22]. When HUVECs were treated with CCCP, migration capacity of HUVECs was markedly weakened (Figure 6(a,b)). Sal B significantly alleviated migration capacity of CCCP-induced HUVECs. MitoTracker Red CMXRos staining demonstrated that mitochondrial activity was prominently damaged by CCCP treatment (Figure 6(c,d)). However, Sal B significantly enhanced mitochondrial activity in CCCP-induced HUVECs. By Flou-4 AM probe, we detected intracellular Ca^2+^ levels. Both in HG- and CCCP-induced HUVECs, intracellular Ca^2+^ levels were markedly weakened compared to controls (Figure 6(e–h)). Sal B treatment prominently increased intracellular Ca^2+^ levels both in HG- and CCCP-induced HUVECs.

### Sal B treatment alleviates apoptosis and mitophagy in CCCP-induced HUVECs

**Figure 7.**
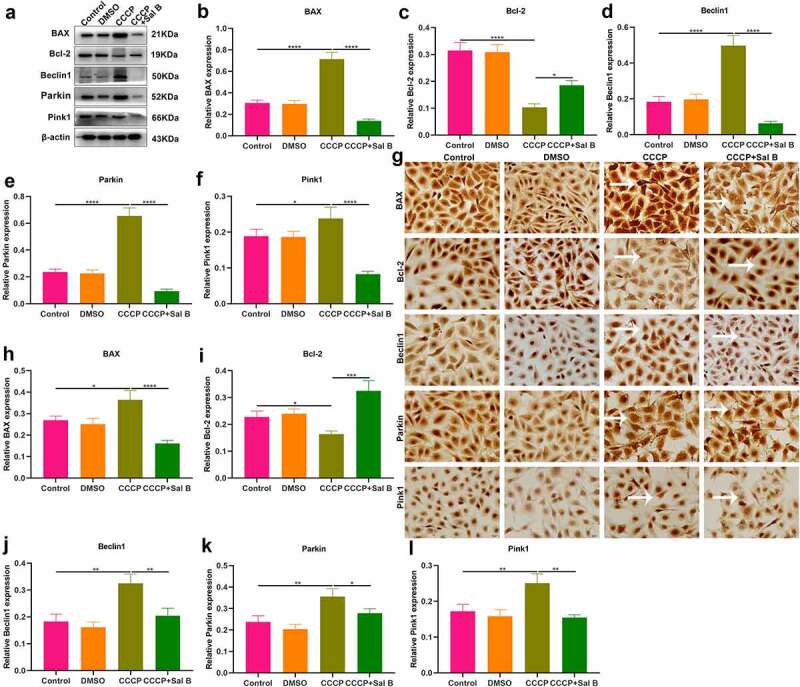
Sal B treatment alleviates apoptosis and mitophagy in CCCP-induced HUVECs. (a–f) Western blot measuring BAX, Bcl-2, Beclin1, Parkin and Pink1 expression in HUVECs of control, DMSO, CCCP and CCCP + Sal B groups. (g–l) IHC staining assessing the expression of BAX, Bcl-2, Beclin1, Parkin and Pink1 in HUVECs of above groups. Scale bar, 20 μm. *P < 0.05; **p < 0.01; ***p < 0.001; ****p < 0.0001. Arrows indicate the abnormalities as claimed.

**Figure 8.**
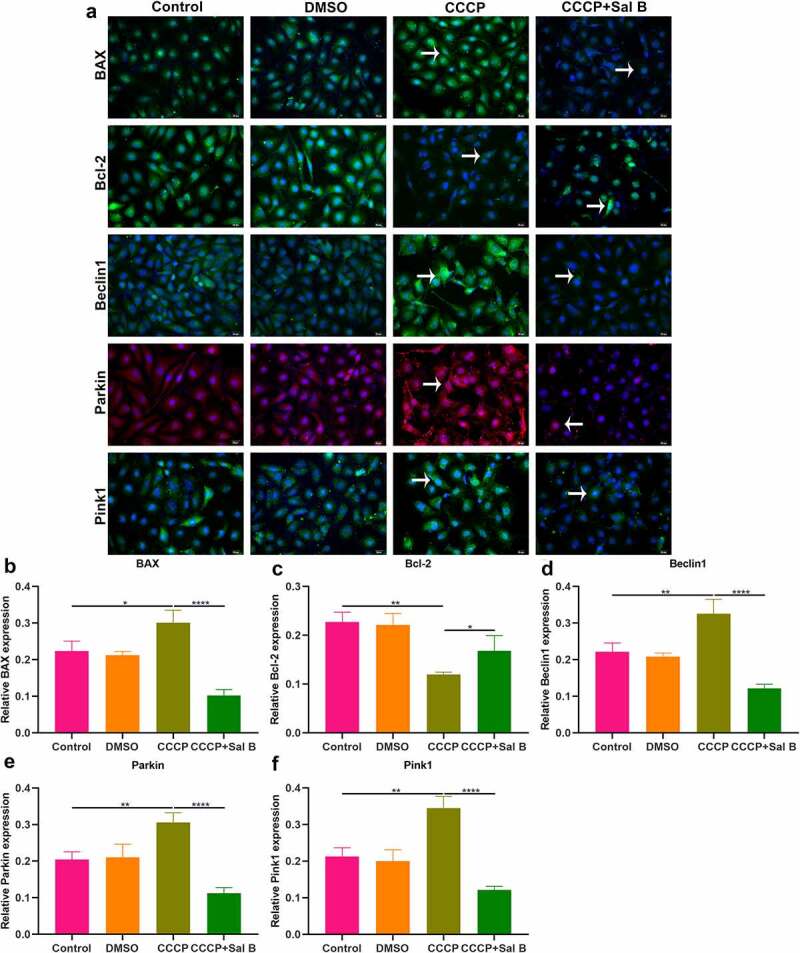
Effects of Sal B treatment on apoptosis and mitophagy in CCCP-induced HUVECs. (a) Representative images of immunofluorescence staining for BAX, Bcl-2, Beclin1, Parkin and Pink1 expression in HUVECs of control, DMSO, CCCP and CCCP + Sal B groups. (b–f) Quantification of BAX, Bcl-2, Beclin1, Parkin and Pink1 expression in HUVECs of above groups. Scale bar, 20 μm. *P < 0.05; **p < 0.01; ****p < 0.0001. Arrows indicate the abnormalities as claimed.

The role of Sal B on apoptosis and mitophagy was further evaluated in CCCP-induced HUVECs. As shown in Western blot results, BAX presented increased expression and Bcl-2 exhibited reduced expression in CCCP-induced HUVECs than controls (Figure 7(a–c)). Nevertheless, Sal B prominently reversed the increase in BAX and the reduction in Bcl-2 in CCCP-induced HUVECs. We also observed the increase in Beclin1, Parkin and Pink1 expression in CCCP-induced HUVECs (Figure 7(d–f)). But Sal B treatment substantially weakened their expression in CCCP-induced HUVECs. IHC staining also showed that BAX expression was distinctly elevated and Bcl-2 expression was significantly reduced in CCCP-induced HUVECs, which was ameliorated by Sal B treatment (Figure 7(g–i)). Moreover, there was increased expression of Beclin1, Parkin and Pink1 in CCCP-induced HUVECs and Sal B treatment markedly alleviated their expression in CCCP-induced HUVECs (Figure 7(j–l)). Immunofluorescence staining confirmed that Sal B treatment prominently alleviated apoptosis in CCCP-induced HUVECs by reducing BAX expression and increasing Bcl-2 expression (Figure 8(a–c)). Also, mitophagy was markedly weakened by Sal B treatment in CCCP-induced HUVECs through decreasing the expression of Beclin1, Parkin and Pink1 (Figure 8(d–f)).

## Discussion

Diabetes may increase the risk of development of cardiovascular diseases [8]. Chronic hyperglycemia can directly result in injury of endothelial cells, and endothelial dysfunction serves as a key initiator, which induces diabetic cardiovascular events [23]. Hence, to develop efficient drugs are regarded as promising strategies to improve diabetic endothelial function. Our study found that Sal B could alleviate diabetic endothelial and mitochondrial dysfunction via down-regulating apoptosis and mitophagy of endothelial cells both in db/db mice and HG- and CCCP-induced HUVECs.

This study established diabetic db/db mice characterized by hyperlipidemia, hyperglycemia, hyperinsulinemia, and insulin resistance. Treatment of 50 mg/kg Sal B for 6 weeks significantly ameliorated hyperlipidemia implied by reduced serum TC, TG and LDL-C levels and increased serum HDL-C levels, hyperglycemia implied by decreased FBG levels, hyperinsulinemia implied by lowered FINS levels, and insulin resistance implied by reduced HOMA-IR and QUICKI values. It has been found that Sal B may ameliorate glucose metabolism and insulin sensitivity in cirrhotic rats [24]. Hyperglycemia with endothelial abnormalities acts as a determining factor of chronic diabetes complications [6]. Moreover, diabetic patients with hypertriglyceridemia and insulin resistance present higher risk of cardiovascular diseases [25]. Endothelial dysfunction serves as an independent prognosticator of cardiovascular diseases [26]. According to H&E, AB, elastic fiber, Masson, and reticular fiber staining, vascular endothelial dysfunction was found in thoracic aorta of db/db mice. Sal B significantly ameliorated diabetes-induced vascular endothelial dysfunction in db/db mice. As described, Sal B may protect against oxLDL- [18], angiotensin II- [27] and HG- [28] induced endothelial dysfunction. Hence, Sal B presented the well therapeutic effects against diabetic endothelial dysfunction.

HG is the main link between diabetes and relevant vascular complications [6]. Thus, we constructed HG-induced HUVECs. Both diabetic thoracic aorta and HG-induced HUVECs presented endothelial and mitochondrial dysfunction. Mitochondrial dynamics and mitophagy are key processes for modulating mitochondrial homeostasis [29]. Morphological and functional alterations of mitochondria exert crucial roles in Diabetes-induced endothelial dysfunction [30]. Mitophagy is an important mechanism to maintain the quality of mitochondria. It is a complex process involving interplay between mitochondria and autophagy mechanisms, and damaged or unwanted mitochondria can be selectively removed. Accumulating evidence demonstrates that alleviating endothelial mitophagy can protect endothelial cells from hyperglycemia. For instance, mesenchymal stem cells alleviate hyperglycemia-mediated endothelial damage via Pink1/Parkin-independent mitophagy [31]. By detection of apoptosis markers (BAX and Bcl-2) and mitophagy markers (Beclin1, Parkin and Pink1), we observed that Sal B markedly ameliorated apoptosis and mitophagy both in diabetic thoracic aorta and HG-induced HUVECs. It has been found that Sal B may alleviate myocardial ischemic damage through enhancing mitophagy and inactivating NLRP3 inflammasome [32]. Moreover, it may protect against oxLDL- and HG-induced endothelial dysfunction through downregulation of ROCK1-modulated mitophagy and apoptosis [18]. Defective mitochondrial calcium presents the correlation to the decreased endothelial bioavailability of nitric oxide, leading to defects in endothelial-dependent vasodilation, which is a key feature of diabetic endothelial dysfunction [33,34]. Changes in the structure and function of endothelial cells are the pathological basis of many cardiovascular diseases, especially diabetes [33,34]. HG is a common cause of endothelial dysfunction. In this study, Sal B could alleviate HG-induced apoptosis and mitochondrial dysfunction in endothelial cells, thereby improving vascular endothelial dysfunction. Therefore, Sal B might delay the progression of cardiovascular diseases, reduce its mortality, and improve the quality of life of patients.

By treating the cells with the uncoupling agent CCCP, mitochondria can be depolarized to simulate the mitochondrial damage state under experimental conditions [35,36]. CCCP results in a sharp drop in the membrane potential across the inner mitochondrial membrane, which is required for protein import through the translocator complex in the inner mitochondrial membrane [35,36]. Pink1 can selectively recruit Parkin to the mitochondria in damaged cells, resulting in uncoupling. Even without mitochondrial membrane depolarization, Pink1 avoids the degradation pathway and constitutively recruits Parkin to the mitochondria, causing mitophagy in otherwise healthy mitochondria [35,36]. Therefore, CCCP induces mitochondrial damage through activating Pink1/Parkin-mediated mitophagy and decreasing mitochondrial membrane potential [35,36]. Sal B significantly ameliorated CCCP-induced mitophagy in HUVECs by up-regulating the expression of Pink1, Parkin and Beclin1. Moreover, our data suggested that Sal B increased intracellular Ca^2+^ levels both in HG- and CCCP-induced HUVECs. Furthermore, Sal B treatment enhanced migration and mitochondrial activity in HG- and CCCP-induced HUVECs. Taken together, our findings revealed that Sal B treatment may protect against diabetes-induced endothelial and mitochondrial dysfunction.

## Conclusion

Collectively, this study demonstrated that Sal B protected against hyperglycemia-induced endothelial dysfunction. In thoracic aorta of db/db mice, HG- and CCCP-induced HUVECs, Sal B alleviated apoptosis and mitophagy of endothelial cells. Moreover, Sal B markedly improved migration, mitochondrial activity and intracellular Ca^2+^ levels both in HG- and CCCP-induced HUVECs. Hence, Sal B possessed the potential as a promising therapeutic agent against diabetic endothelial and mitochondrial dysfunction, which was involved in the down-regulation of apoptosis and mitophagy of endothelial cells.

## Data Availability

The datasets analyzed during the current study are available from the corresponding author on reasonable request.
